# Supplementary material for: CDH13 Is Associated with Cellular Viability After Exposure to Ionizing Radiation Using Genome-Wide Screening
Source: Int J Mol Sci. 2026 Jul 30;27(15):6826. doi: 10.3390/ijms27156826 (PMC13467385; doi:10.3390/ijms27156826)
Supplement: Supplementary file 1 [file ijms-27-06826-s001.zip › Supplementary Figures.pdf]

Supplementary Figure S1

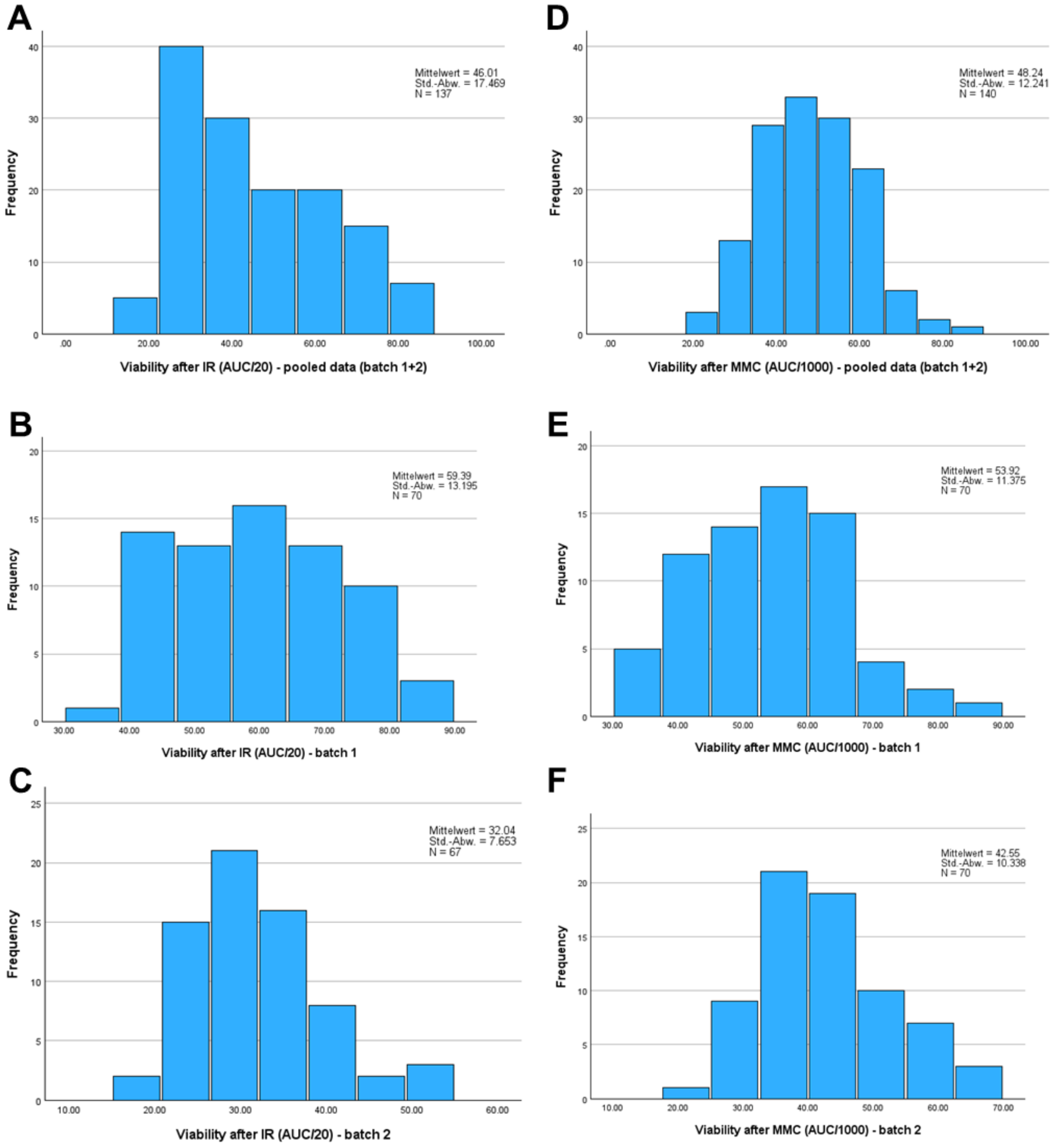

Supplementary Figure S1: Phenotype distribution

(A) through (C) show the viability after IR treatment, whereas (D) through (F) show the viability after MMC treatment. More precisely, the x-axes show the viability after MMC/IR treatment as indicated by the AUC values of the viability curves of the cell lines. For illustration purposes, AUC/20 was used to show the viability after IR treatment and AUC/1000 for MMC. The y-axes show the respective frequencies. (A) and (D) respectively show the values for the pooled data

(batch 1 + 2), (B) and (E) only for batch 1 and (C) and (F) only for the batch 2. The figure also includes the sample sizes as well as the mean values and standard deviations (SD).

Supplementary Figure S2

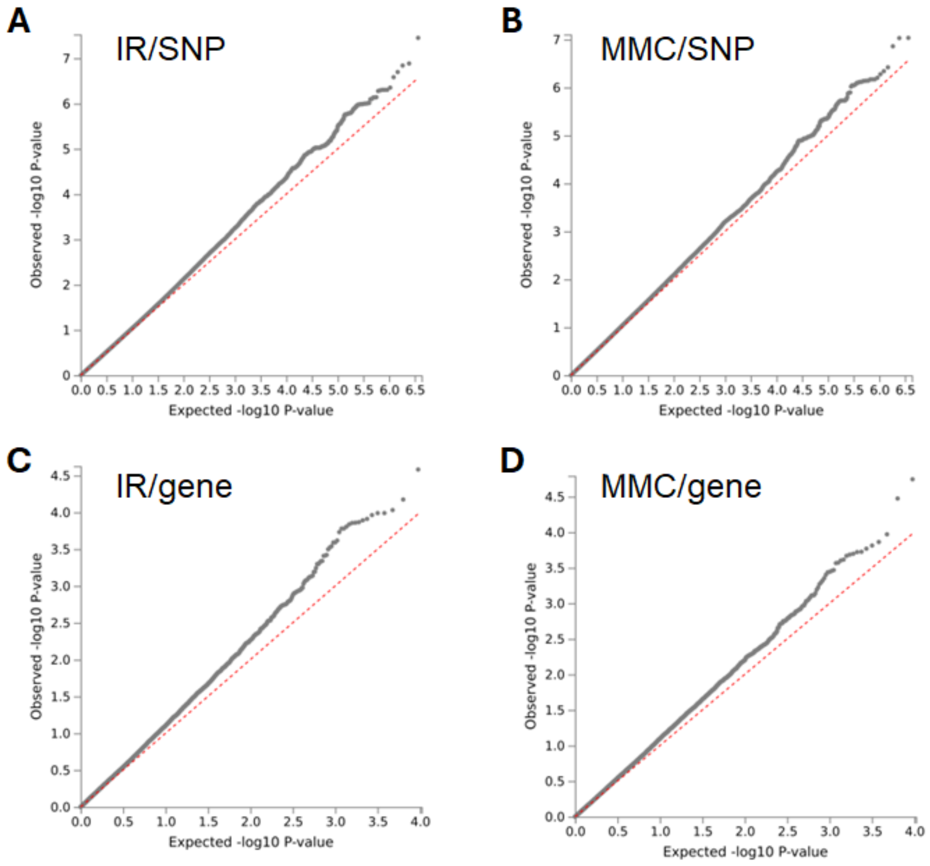

Supplementary Figure S2: Quantile-quantile plots from the GWAS of IR-AUCs and MMC-AUCs for SNP the SNP-based and gene-based meta-analyses
